# The expression and activity of β-catenin in the thalamus and its projections to the cerebral cortex in the mouse embryo

**DOI:** 10.1186/1471-2202-13-20

**Published:** 2012-02-23

**Authors:** Thomas Pratt, John W Davey, Tomasz J Nowakowski, Casey Raasumaa, Konrad Rawlik, Derek McBride, Michael Clinton, John O Mason, David J Price

**Affiliations:** 1 Genes and Development Group, Centre for Integrative Physiology, School of Biomedical Sciences, University of Edinburgh, Hugh Robson Building, George Square, Edinburgh EH8 9XD, Scotland, UK; 2 Division of Developmental Biology, The Roslin Institute and R(D)SVS, University of Edinburgh, Easter Bush, Midlothian EH25 9RB, Scotland UK

**Keywords:** β-catenin, Netrin-1, Thalamus, Growth cones, mRNA, BAT-gal

## Abstract

**Background:**

The mammalian thalamus relays sensory information from the periphery to the cerebral cortex for cognitive processing via the thalamocortical tract. The thalamocortical tract forms during embryonic development controlled by mechanisms that are not fully understood. β-catenin is a nuclear and cytosolic protein that transduces signals from secreted signaling molecules to regulate both cell motility via the cytoskeleton and gene expression in the nucleus. In this study we tested whether β-catenin is likely to play a role in thalamocortical connectivity by examining its expression and activity in developing thalamic neurons and their axons.

**Results:**

At embryonic day (E)15.5, the time when thalamocortical axonal projections are forming, we found that the thalamus is a site of particularly high β-catenin mRNA and protein expression. As well as being expressed at high levels in thalamic cell bodies, β-catenin protein is enriched in the axons and growth cones of thalamic axons and its growth cone concentration is sensitive to Netrin-1. Using mice carrying the β-catenin reporter *BAT-gal *we find high levels of reporter activity in the thalamus. Further, Netrin-1 induces *BAT-gal *reporter expression and upregulates levels of endogenous transcripts encoding β-actin and L1 proteins in cultured thalamic cells. We found that β-catenin mRNA is enriched in thalamic axons and its 3'UTR is phylogenetically conserved and is able to direct heterologous mRNAs along the thalamic axon, where they can be translated.

**Conclusion:**

We provide evidence that β-catenin protein is likely to be an important player in thalamocortcial development. It is abundant both in the nucleus and in the growth cones of post-mitotic thalamic cells during the development of thalamocortical connectivity and β-catenin mRNA is targeted to thalamic axons and growth cones where it could potentially be translated. β-catenin is involved in transducing the Netrin-1 signal to thalamic cells suggesting a mechanism by which Netrin-1 guides thalamocortical development.

## Background

The adult thalamus is a complex structure in the centre of the brain, comprising clusters of functionally related cells organised into a large number of nuclei. Thalamic nuclei form precise reciprocal connections with their targets in the cerebral cortex providing it with most of its sensory innervation *via *thalamocortical axons. In mice axons grow from the thalamus into the ventral telencephalon at around embryonic day (E)12-13 and then on to the cerebral cortex which they first reach at around E13-14 [1-7]. The development of the thalamus and its connections relies on intercellular communication mediated by secreted signalling proteins including Wnt, Slit, and Netrin proteins [8-19].

Wnt signalling components are expressed in complex patterns in the developing thalamus itself and in the territory encountered by thalamocortical axons. Wnt signalling is known to be important for thalamic development as targeted disruption of Wnt proteins or their receptors result in severe thalamic development and connectivity defects [9,20-23]. β-catenin is an intracellular protein that can affect both cytoskeletal dynamics involved in cell motility and gene expression in the nucleus in response to extracellular signals including Wnt proteins [24-35]. An intriguing feature of the adult thalamus is the expression of high levels of β-catenin protein. In fact the thalamus is unique within the adult CNS in having sufficiently high levels of nuclear β-catenin to be easily detectable with immunohistochemistry and β-catenin mediated TCF/LEF transcription plays a key role in defining the electrophysiological properties of thalamic cells [36,37]. While the manipulation of β-catenin activity has provided insights into the function of β-catenin in neural progenitor cells there are as yet no tractable transgenic models which allow the role of β-catenin to be studied in post-mitotic neurons [38-43].

In this study we address the role of β-catenin in the thalamus and its axons at the time the thalamocortical tract is starting to form. First we use in situ hybridisation, immunohistochemistry, and a *BAT-gal *reporter transgene to show that β-catenin is expressed at high levels both in cell bodies and in axons in the developing thalamus and that β-catenin mediated transcription is very active in thalamic cells at this time. Netrin-1 is known to be a key regulator of thalamocortical development [14,15]. While the relationship between the Wnt response and β-catenin is well established, β-catenin's relationship with Netrin-1 is not, so we next used in vitro assays to show that Netrin-1 treatment causes an increase in levels of β-catenin protein in thalamic growth cones and induces β-catenin dependent gene expression in thalamic cells. Local translation of mRNAs in growth cones is a well established mechanism to facilitate rapid changes in growth cone protein levels in response to guidance cues including Netrin-1 so we hypothesised that thalamic axons might contain β-catenin mRNA [44-56]. We performed an unbiased screen for mRNAs present in thalamic axons, recovered β-catenin transcripts at high frequency and identified other transcripts in thalamic axons. Using a combination of in situ hybridisation, GFP reporter transgenes, and quantitative RT-PCR we showed that β-catenin mRNA is enriched in thalamic axons and sequence elements in its highly conserved 3'UTR enhance protein expression along the thalamic axon..

## Results

### Expression of β-catenin in the developing thalamus

**Figure 1:**
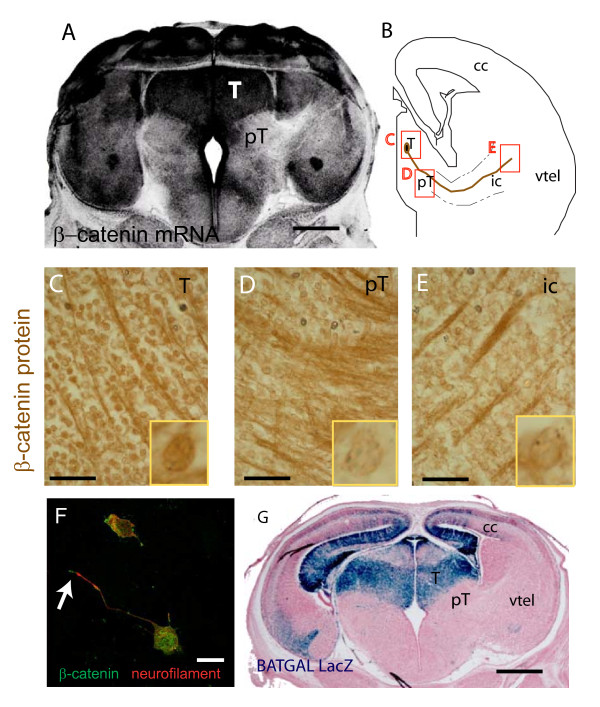
**β-catenin expression and activity in the developing thalamocortical tract at E15.5**. (**A**) In situ hybridisation for β-catenin mRNA on coronal section of E15.5 mouse embryo (black signal). (B-E) DAB immunohistochemistry for β-catenin protein. (**B**) Diagram indicating boxed areas shown at high magnification in (C-E) and illustrating a thalamic cell body in the thalamus (T) with its axon growing through the prethalamus (pT) and into the internal capsule (ic) and towards the cerebral cortex (cc). Higher magnification of (**C**) thalamus, (**D**) prethalamus, and (**E**) internal capsule, insets show a blown-up cell body. Note that although axonal staining is strong in all areas cell body staining is relatively much stronger in the thalamus. (**F**) β-catenin/neurofilament double immunofluorescence on a cultured thalamic neuron with arrow indicating a growth cone. (**G**) LacZ histochemistry on a BAT-gal reporter embryo. Scale bars: A&G, 500 μm; C-E, 100 μm; F, 10 μm.

To assess the role of β-catenin in thalamocortical development we first studied the localisation of β-catenin mRNA and protein in the thalamus at the time when it is projecting axons. In situ hybridisation for β-catenin at E15.5 revealed higher mRNA levels in the thalamus compared to much lower levels in adjacent prethalamus and ventral telencephalon (Figure 1A). Next we used immunohistochemistry to examine the cellular distribution of β-catenin protein in thalamic cells and their axons. Within the thalamus there are numerous heavily stained cell bodies and axon fascicles (Figure 1C, inset shows a blow-up of a cell body). In the prethalamus (Figure 1D) and internal capsule (Figure 1E) of the same section, axon fascicles are heavily stained but cell bodies are much more weakly stained (insets in Figure 1D, E show blow-ups of cell bodies in these areas). Immunofluorescence on cultured thalamic neurons shows that there are relatively high levels of β-catenin in the cell body and at the growth cone with lower levels in the intervening axon (Figure 1F, arrow points to growth cone).

The *BAT-gal *reporter transgene comprises multiple TCF/LEF binding sites coupled to a bacterial *LacZ *gene and provides a convenient readout of β-catenin mediated transcription [57]. At E15.5 the *BAT-gal *transgene reports at very high levels in the thalamus and much lower levels in the prethalamus, ventral telencephalon, and cerebral cortex through which thalamic axons navigate (Figure 1G).

In conclusion, at the time the thalamus is projecting axons towards the cerebral cortex at E15.5, thalamic cells contain high levels of β-catenin mRNA and protein and are the site of particularly vigorous β-catenin mediated transcription.

### Netrin-1 induces changes in mRNAs and in β-catenin protein levels in thalamic cells

**Figure 2:**
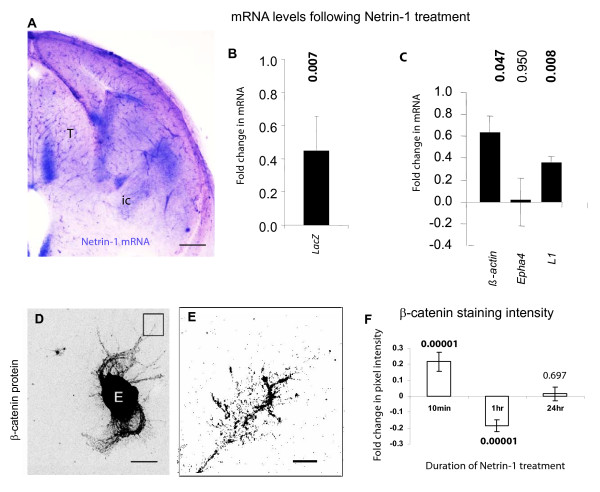
**Effect of Netrin-1 on thalamic cells and axons**. (**A**) In situ hybridisation for Netrin-1 mRNA (purple signal) on a coronal E14.5 forebrain section. (**B**,**C**) mRNA levels measured after 90 minutes exposure to Netrin-1 expressed relative to untreated controls. (B) *LacZ *mRNA levels in cultured *BAT-gal *thalamic explants. (**C**) *β-actin*, *Epha4*, and *L1 *mRNA levels in cultured thalamic explants. Transcript levels were measured using qRT-PCR and normalised to *GAPDH *with n = 3 for each condition. (**D**) Cultured thalamic explant immunostained for β-catenin with higher magnification of a growth cone in box shown in (**E**). (**F**) Growth cone β-catenin staining pixel intensity measured after 10 minutes, 1 hour, or 24 hours exposure to Netrin-1 expressed relative to untreated controls. For each condition values represent means for n = 84-95 growth cones randomly selected from 3 independent cultures each including thalamic tissue from several embryos Students *t*-test p values for ± Netrin-1 comparion indicated above histogram bars at each time-point. Scale bars: A = 200 μm; D, 100 μm; E, 5 μm.

Netrin-1 mRNA is present both in the thalamus, where thalamocortical cell bodies reside, and in the internal capsule, which thalamic axons grow through (Figure 2A), so both thalamic cells and their axons are likely to encounter Netrin-1 protein.

First, we hypothesised that the high levels of β-catenin mediated transcription we observed in the thalamus of *BAT-gal *embryos (Figure 1G) are contributed to by Netrin-1. We used qRT-PCR to measure levels of *LacZ *mRNA in thalamic cultures prepared from *BAT-gal *embryos. As shown in Figure 2B there was a significant increase in *LacZ *mRNA in cultures exposed to Netrin-1 for 90 minutes compared to untreated controls, showing that Netrin-1 can induce β-catenin mediated transcription in thalamic cells. L1 regulates thalamocortical axon fasciculation in the internal capsule and L1 mRNA is upregulated in response to β-catenin signalling in other systems, expression of β-actin can be regulated by Netrin-1 in *Xenopus *retinal growth cones, and Epha4 is required for ordered thalamocortcial axon navigation in the internal capsule and has not been linked with either β-catenin or Netrin-1 function [58-61]. We found significant increases in the thalamic levels of *β-actin *and *L1 *mRNAs but not *Epha4 *mRNA following Netrin-1 treatment for 90 minutes (Figure 2C).

We next asked whether Netrin-1 treatment affects growth cone β-catenin. In order to monitor the response of thalamic cells to Netrin-1 we employed a culture system in which thalamic explants were cultured on glass coverslips and allowed to extend axons. An example of a cultured thalamic explant, immunostained with β-catenin, is shown in Figure 2D. Cultures were exposed to Netrin-1 (400 ng/ml), a concentration that has previously been shown to stimulate thalamic axon growth [14]. Thalamic axons were exposed to Netrin-1 for various time intervals (10 minutes, 1 hour, or 24 hours) and processed for β-catenin immunofluorescence. An example of a growth cone stained for β-catenin is shown in Figure 2E. The total pixel intensity of β-catenin indirect immunofluorescence was measured for each growth cone and values for growth cones treated with Netrin-1 were then normalised against untreated control growth cones to show fold changes in the β-catenin signal (Figure 2F). Ten minutes of exposure to Netrin-1 caused a 22% increase in β-catenin signal. After 1-hour exposure to Netrin-1, the β-catenin signal in the growth cones was reduced by 19%. One day after adding Netrin-1 values were similar to those in untreated growth cones. This experiment demonstrates rapid and dynamic fluctuations in growth cone β-catenin concentration in response to the thalamocortical guidance cue Netrin-1.

### Identification of β-catenin mRNA in thalamic axons

Our data so far suggest a possible role for β-catenin in thalamic axon navigation. The rapid changes in β-catenin protein levels in growth cones exposed to Netrin-1 suggest rapid β-catenin protein synthesis, perhaps followed by its degradation or movement away from the growth cone, in response to guidance cues. We next turned our attention to the distribution of β-catenin mRNA in the thalamocortical system, particularly in the axons and growth cones where mRNAs might be locally translated. As shown in Figure 1A, β-catenin mRNA is expressed at high levels by thalamic cells so we were interested to know whether the β-catenin transcript is present in axons and, to place our findings in context, how the distribution of β-catenin mRNA related to that of other transcripts. We therefore isolated and analysed a set of mRNAs found in the axons of embryonic thalamic neurons. In brain sections thalamic axons are closely associated with other cells and axons so we performed this analysis in cultures where thalamic axons can be unambiguously identified and isolated.

**Figure 3:**
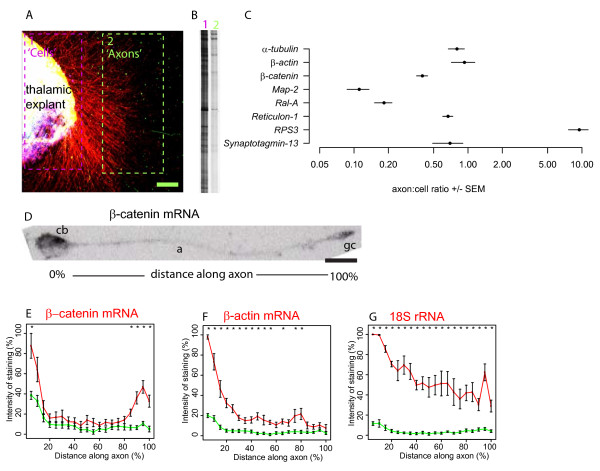
**Distribution of mRNAs in cultured thalamic cells, explants, and their axons**. (**A**) Photomicrograph of cultured thalamic explant (on left) with axons growing from it stained with an antibody against neurofilament. Broken lines outline the regions cut out to obtain samples for RNA extraction. (**B**) A portion of a gel showing PCR amplified products from mRNAs taken from area 1 'Cells' (left lane) and area 2 'Axons' (right lane). (**C**) Axon:Cell ratios for 8 mRNAs measured by qRT-PCR. Each point shows mean ± SEM for 3 samples. (**D**) in situ hybridisation for *β-catenin *mRNA (black signal) on a dissociated thalamic neuron. (E-G) Quantification of hybridisation signal density for (**E**) *β-catenin *mRNA, (**F**) *β-actin *mRNA, and (**G**) *18S *rRNA with antisense probe signal in red and background signal in green. Lines used for densitometric analysis were drawn along the axon connecting the cell body (cb- 0% along the line) and growth cone (gc- 100% along the line) as indicated in (**D**). The *β-catenin *antisense probe (n = 12) and *β-actin *antisense probe (n = 11) are compared against a *β-catenin *sense probe (n = 15), whereas the *18S *oligoprobe (n = 6) is compared against a Scrambled oligoprobe (n = 7). The sense and scrambled control probes are used to define background signal levels. An asterisk above a data point indicates significant difference from background (Student's t-test, *p *< 0.05). Note that each transcript has a distinct distribution profile along the axon. Abbreviations: cell body (cb); growth cone (gc); axon (a). Scale bars: A, 100 μm; E, 10 μm.

Thalamic explants were placed in culture for 3 days allowing long axons to grow out (Figure 3A) and then RNA was prepared either (1) from thalamic explants which comprise cell bodies, axons, and growth cones or (2) from axons and growth cones alone (dissected regions 1 'cells' & 2 'axons' indicated in Figure 3A). Randomly-primed cDNA was generated from these two RNA samples and Figure 3B shows a portion of a gel separating the amplified products.

Thalamic axonal cDNA was used to generate a plasmid library from which clones were randomly selected for sequencing. Sequence data was recovered from 87 clones. BLAST analysis of these sequences against the mouse genome sequence revealed that the vast majority of them (84%) mapped to transcribed regions (introns and exons of protein coding genes and ribosomal RNA). The remaining 16% of sequences mapped to regions annotated in the ENSEMBL database as non-genic and we did not investigate them further although, given that newly transcribed regions of the genome are still being discovered, it is quite possible that they have functional significance [62].

Of the 73 clones that mapped to transcribed regions, 30% contained transcripts for mitochondrial 16S rRNA, a component of the mitochondrial ribosome encoded by the mitochondrial genome [63], whose presence in axons is predictable because they contain mitochondria. Most of the remaining 51 clones mapped to exons of protein coding transcripts. Table 1 lists protein coding genes for which cloned sequences mapped to exons. Some genes were represented by more than one clone, notably *Ribosomal protein S3 *(*RPS3*) (6 clones), *β-catenin *(5 clones) and *β-actin *(4 clones). Also in Table 1 are clones recovered from the axonal library that mapped to predicted intronic regions (*Wnt2b*, *Neurexin 1α*, *reticulon 1*, *DNA polymeraseβ*, *muskelin1*, *Coiled-coil domain containing 11*). These might be alternatively spliced exons not in the ENSEMBL database (highly likely for *Reticulon-1 *mRNA which we independently confirmed to be in thalamic axons using qRT-PCR- see Figure 3C) or might correspond to distinct overlapping genes.

**Table 1:** Identities of thalamic axonal mRNAs.

ENSEMBL ID	*MGI gene symbol *(*Full name*)	**Location of recovered clone within gene**.
ENSMUSG00000000751	*Rpa1 *(*replication protein A1*)	Exon 5'UTR

		

ENSMUSG00000039643	*Npm1 *(*Nucleophosmin 1*)	Exon ORF

ENSMUSG00000004032	*Gstm5 *(*glutathione S-transferase, Mu 5*)	Exon 3'UTR

ENSMUSG00000021643	*Serf1 *(*small EDRK-rich factor 1*)	Exon 3'UTR

ENSMUSG00000028248	*Sfrs 18 *(*serine/arginine-rich splicing factor 18*)	Exon 3'UTR

ENSMUSG00000003660	*Snrnp200 *(*small nuclear ribonucleoprotein 200 kDa (U5)*)	Exon 3'UTR

ENSMUSG00000005312	*Ubqln1 *(*Ubiquilin 1*)	Exon 5'UTR

ENSMUSG00000005873	*Reep5 (receptor expression- enhancing protein 5*)	Exon 5'UTR

ENSMUSG00000006932	*Ctnnb1 *(*β-catenin*)	Exon 3'UTR

ENSMUSG00000008859	*Rala (v-ral simian leukemia viral oncogene homolog A*)	Exon ORF

ENSMUSG00000027220	*Syt13 *(*Synaptotagmin 13*)	Exon 3'UTR

ENSMUSG00000028961	*Pgd *(*6-phosphogluconate dehydrogenase*)	Exon 5'UTR

ENSMUSG00000029580	*Actb *(*β-actin*)	Exon 5'UTR

ENSMUSG00000030744	*Rps3 *(*Ribosomal protein S3*)	Exon 5'UTR

ENSMUSG00000036693	*Nop14 *(*NOP14 nucleolar protein homolog (yeast)*)	Exon ORF

ENSMUSG00000038871	*Bgpm *(*2,3-bisphosphoglycerate mutase*)	Exon 3'UTR

ENSMUSG00000040225	*Bat2l2 *(*HLA-B associated transcript 2-like 2*)	Exon ORF

ENSMUSG00000048120	*Entpd1 *(*ectonucleoside triphosphate diphosphohydrolase 1*)	Exon 3'UTR

ENSMUSG00000021087	*Rtn1 *(*Reticulon 1*)	Intron 1-2/7 (12989 bp)

ENSMUSG00000024109	*Nrxn1 *(*Neurexin 1α*)	Intron 9-10/17 (23196 bp)

ENSMUSG00000031536	*PolB *(DNA polymeraseβ)	Intron 10-11/14 (2779 bp)

ENSMUSG00000025609	*Mkln1 *(*muskelin 1*),	Intron 12-13/18 (11278 bp)

ENSMUSG00000027840	*Wnt2b*	Intron 4-5/5 (3807 bp)

ENSMUSG00000035394	*Ccdc11 *(*Coiled-coil domain containing 11*)	Intron 7-8/9 (27462 bp)

ENSEMBL identifier, MGI gene symbol and common name or description are given for each mRNA along with whether the sequence recovered from the thalamic axon cDNA library mapped to an exon [broken down into 5' or 3' untranslated region (UTR) or open reading frame (ORF)] or an intron [identified by the flanking exons/total number of exons in that gene and the intron size].

In conclusion, our unbiased screen recovered β-catenin mRNA from thalamic axons. The high frequency of β-catenin clone recovery is suggestive of high abundance in thalamic axons. Tellingly, the recovery frequency was similar to that for β-actin mRNA, which has well established function in axon navigation in other systems [59]. The wide range of physiological functions encoded by the other thalamic axonal mRNAs listed in Table 1 mirrors that of mRNAs found in mouse retinal and cortical axons [64,65].

### Localisation of *β-catenin *mRNA in the axon

In order to shed light on the distribution of transcripts between the cell body and the axon we performed a careful quantitative analysis using qRT-PCR to calculate the ratio of β-catenin mRNA in thalamic axons to that in the thalamic explants from which the axons project (the axon/cell ratio in Figure 3C). To place our findings in context we included five other transcripts recovered in our screen (α-tubulin, β-actin, Map-2, Ral-A, reticulon-1, RPS3, and synaptotagmin-13) and *α-tubulin *and *Map2 *which are known to be enriched or depleted in the axonal compartment in other systems [66-68]. *18S *rRNA is a commonly used loading control for qRT-PCR experiments and is found in axons. Transcript levels were normalised to 18S rRNA levels in their own compartment. This ratio allowed us to assess the relative distribution of each transcript between axons and cells in the explant. The wide variation in axon:cell transcript ratios argues that the distribution of these transcripts between axon and cell body compartments is unlikely to be accounted for by a common mechanism, for example passive diffusion from soma to axon, as this would predict a relatively constant ratio. Critically, a high axon:cell ratio suggests that a transcript is actively targeted to the axonal compartment and consistent with this *Map2 *mRNA (which is targeted to the somato-dendritic compartment in other systems) had the lowest ratio while *β-actin *and *α-tubulin *mRNAs (which are axonally targeted in other systems) had ratios about 10-fold higher (note the log-scale in X-axis of Figure 3C) which are comparable to those of *β-catenin*, *Reticulon-1*, and *synaptotagmin-13*. The greatest axonal enrichment was seen in the mRNA encoding the ribosomal protein RPS3.

To determine the location of *β-catenin *mRNA within the growing thalamic axons we used in situ hybridisation on cultured E15.5 thalamic cells. An example of a thalamic neuron stained for *β-catenin *mRNA is shown in Figure 3D where strong staining is apparent in the cell body and in the growth cone with weaker staining in the intervening axon. Densitometric analysis was carried out for *β-catenin *mRNA (Figure 3E). The upper red line in Figure 3E shows quantification of the *β-catenin *in situ signal along thalamic axons from their cell bodies to their growth cones. It shows that the signal is highest in the cell bodies and the growth cones (left and right hand end of the trace respectively) and resembles the distribution of β-catenin protein (Figure 1F). As a control the signal was measured in thalamic neurons reacted with a sense *β-catenin *DIG labelled RNA transcript (lower green line in Figure 3E). The anti-sense signal is significantly above background in the cell body and at the end of the axon indicating that *β-catenin *mRNA is specifically enriched towards the growth cone. An identical analysis of *β-actin *mRNA (Figure 3F) and 18S rRNA (Figure 3G) showed them to be more evenly distributed along the length of the axon*. β-catenin *mRNA is therefore subject to idiosyncratic positioning within the thalamic axon relative to at least two other axonal transcripts.

### Sequence and functional analysis of the β-catenin 3'UTR

**Figure 4:**
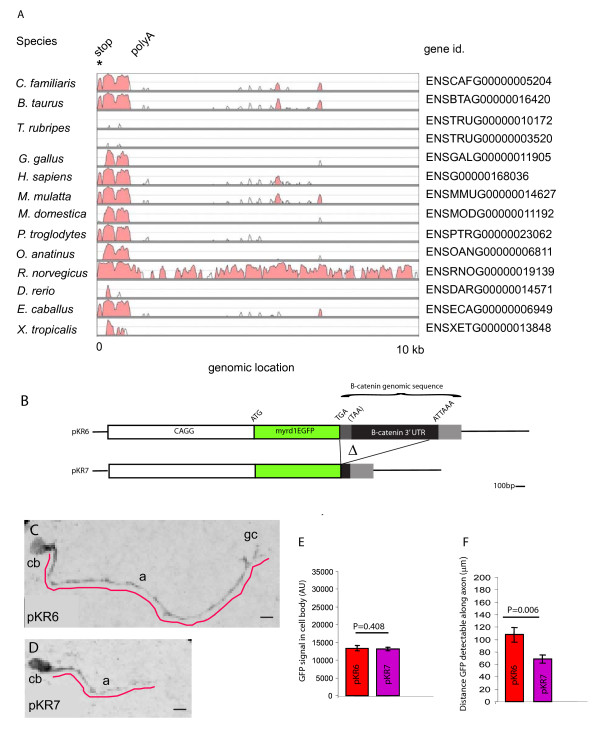
**Sequence and functional analysis of the Β-catenin 3'UTR'.** (A) Evolutionary conservation of the β-catenin 3'UTR in vertebrates. Individual plots show conservation between the indicated species and the mouse based on comparisons between genomic sequence starting at the *β-catenin *stop codon (asterisk) at the 5' limit of the 3'UTR and continuing 10 kb in the 5' direction encompassing the entire 3'UTR and downstream untranscribed sequence. The position of the polyadenylation signal in the mouse sequence, which approximates to the 3' limit of the 3'UTR, is marked poly A. Conservation score calculated as an averaged windowed identity (window size = 100 bp). Curves only show conservation score range 50-100%. Position indicates kilo bases from stop codon in mouse *β-catenin*. (Alignments calculated with M-LAGAN). (B-F) *β-catenin *3'UTR directs GFP expression to the axon. (**B**) Diagram of GFP plasmid reporter constructs. CAGG promoter (unfilled box) *myrd1EGFP *cDNA (green box). pKR6: The myrd1EGFP stop codon (TGA) attached to *β-catenin *genomic sequence spanning from just 3'to the translation stop codon (TAA), through the 3'UTR (black) to approximately 300 bp 5'of the polyadenylation signal sequence (ATTAAA). pKR7 is identical to pKR6 except that most of the 3'UTR is deleted. (**C**, **D**) Examples of thalamic neurons electroporated with GFP reporter constructs and cultured for 2 days, GFP immunostaining gives black signal in these images (**C**) pKR6, (**D**) pKR7. Neurons are growing within a tissue explant so cell bodies (cb) and axons (a) are only visible if GFP is expressed in them. The red lines illustrate lines drawn to measure the distance GFP signal could be detected along an axon. Note that the red line in C extends to a growth cone. (**E**) Densitometric analysis of GFP signal intensity (in arbitrary units (AU)) in cell bodies following transfection with pKR6 and pKR7. (**F**) Distance GFP signal could be traced from the cell body along the axon. Transfecting thalamic explants with pTP6 to express a tau tagged GFP which completely fills axons gave a value for total axon length of 158 ± 14 μm. Mann-Whitney Rank sum test P-values for pairwise comparisons shown above bars, error bars are ± SEM. GFP values were taken from 84 and 30 (pKR6) and 116 and 30 (pKR7) cell bodies and axons respectively or 30 axons (pTP6) randomly selected from 3 independent cultures each including thalamic tissue from several embryos. Scale bars in C, D, 10 μm.

Protein coding mRNAs comprise an open reading frame (ORF) flanked by 5' and 3' UTRs with sequence elements responsible for subcellular localisation frequently residing in the 3'UTR so we next looked for evidence of phylogenetic conservation of β-catenin mRNA 3'UTR sequences. Mouse *β-catenin *genomic sequence starting from the stop codon and extending 10 kb in the 3' (downstream) direction was compared to a variety of equivalent 10 kb vertebrate *β-catenin *genomic sequences using a M-LAGAN alignment algorithm. This revealed regions of striking sequence similarity within placental and marsupial mammalian species (*C. familiaris*, *B. Taurus*, *H. sapiens*, *M. mulatta*, *P. troglodytes*, *M. domestica*, *R*. *norvegicus*, *E. caballus*, &*O. anatinus*) with three closely adjacent peaks of conservation (Figure 6A). This conservation extended to birds (*G. gallus*) and, although diminished, two peaks were still apparent in fish (*D. rerio & T. rubripes *- note that *T. rubripes *has two *β-catenin *genes). Notably this conserved region spanned ~1 kb from the translation stop codon (asterisk in Figure 4A) to the polyadenylation signal (PolyA in Figure 4A) coinciding with the *β-catenin *3'UTR. This conservation is almost as high as that seen in the β-catenin protein coding ORF (not shown) suggesting that functionally important sequences reside in the 3'UTR.

We hypothesised that one conserved function of the *β-catenin *3'UTR is to direct protein expression to the axon. To test this hypothesis in thalamic axons we generated a reporter plasmid construct, pKR6 (Figure 4B), comprising a myristoylated destabilised GFP (*myrd1EGFP*) cDNA fused at its 3' end to *β-catenin *genomic sequences encompassing the translation stop codon, 3'UTR, polyadenylation signal, and 300 bp of downstream sequence. Another construct, pKR7 (Figure 4B), was generated with the majority of the 3'UTR deleted leaving only a stub comprising the polyadenylation signal and sequence downstream of the 3'UTR. This is a well established method to report on *de novo *translation because myrd1EGFP protein contains a myristoylation sequence (Myr) to limit its diffusion from the site of translation and has a half-life of only one hour [53]. Transcription was driven by the powerful ubiquitous CAGG promoter [69]. If the hypothesis is correct then the chimeric *GFP:β-catenin 3'UTR *mRNA produced by transcription of pKR6 should be targeted to and translated in axons. Reporter plasmids were introduced into thalamic explants by electroporation and the explants cultured for 2 days. Cultures were then processed for GFP immunofluorescence (examples of images shown in Figure 4C, D). Two parameters were quantified from these images: (1) the GFP fluorescence of the cell body and (2) the distance from the cell body where GFP fluorescence could be found in the axon. Comparison of cell body values showed that pKR6 and pKR7 had indistinguishable GFP levels indicating that the majority of the *β-catenin *3'UTR is not needed for expression within the cell body (Figure 4E). The cell body GFP levels measured for pKR6 and pKR7 are subsaturating as a *myrd1EGFP:SV40 3'UTR *construct in an otherwise identical experiment yielded over 2-fold the cell body GFP values in Figure 4E (data not shown). The *β-catenin *3'UTR contributes to axonal expression of GFP as the pKR6 construct containing the full *β-catenin *3'UTR sequence frequently drove GFP expression all the way along the axon to a growth cone (Figure 4C) and almost twice as far along the axon as the pKR7 construct from which most of the *β-catenin *3'UTR was deleted (Figure 4F). In order to estimate the proportion of the axon these distances correspond to we performed an otherwise identical experiment using pTP6 which expresses a tau-tagged GFP (neither destabilised nor myrisoylated) which efficiently fills thalamic axons [70] giving a value for total axon length of 158 ± 14 μm. This allowed us to calculate that, on average, pKR6 drove detectable GFP signal 67% along the axon compared to 43% for pKR7. These data are not consistent with axonal GFP expression passively reflecting GFP expression levels in the cell body as pKR6 and pKR7 have similar cell body GFP levels but pKR6 drives GFP significantly further along the length of the axon.. In conclusion the 3'UTR of β-catenin mRNA is sufficient to enhance the expression of a heterologous reporter protein in thalamic axons so is presumably able to do the same for endogenous β-catenin protein.

## Discussion

The dual functions of β-catenin in connecting cadherin molecules on the cell surface to the actin cytoskeleton and in regulating TCF/LEF mediated gene expression in response to Wnt signalling in the nucleus make it an interesting candidate for coordinating the development of neural structures and connectivity [71]. While attention has focussed on the importance of Wnt/β-catenin signalling in neural progenitors in the developing thalamus and elsewhere in the brain, the function of β-catenin activity in post-mitotic projecting neurons remains relatively unexplored [9,21,36,37,43]. The developing embryonic thalamus is a site of high levels of TCF/LEF transcription mediated by nuclear β-catenin [20,57] & present study. While a component of thalamic TCF/LEF transcription is likely a response to Wnt signalling, Slit proteins which are also abundant in the thalamus and tissues encountered by its axons can turn on TCF/LEF transcription when sensed by their Robo receptor [8,25]. Our new finding that Netrin-1 can activate TCF/LEF transcription adds to these options.

A key finding in this study is the rapid increase in the levels of β-catenin protein in thalamic growth cones in response to the axon guidance cue Netrin-1. As thalamic axons extend towards the cerebral cortex their growth cones become increasingly distant from the cell body posing a potential logistical problem if all new protein must be translocated from the cell body. A classic mechanism to overcome this is for growth cones to carry mRNAs that can be translated locally [45,46,53,55,56]. As a precedent from another system, *β-catenin *mRNA is found in migrating astrocytic filopodia and hippocampal growth cones where its translation in response to the neurotrophic factor NT3 is dependent on the Cytoplasmic Polyadenylation Element-Binding Protein (CPEBP) binding to discrete elements in its 3'UTR [72,73]. Therefore, while we have not directly addressed whether or not β-catenin is locally translated in thalamic growth cones in the present study, our finding that β-catenin mRNA is present in thalamic axons and that elements in its 3'UTR assist protein expression in the thalamic axonal compartment make it a strong possibility.

## Conclusions

We provide evidence that β-catenin mRNA and protein are expressed at high levels in mouse embryonic thalamic cells and their axons at the time connections are being formed with the cerebral cortex. We find that Netrin-1 induces β-catenin mediated transcriptional activity in thalamic cells and induces rapid changes in the growth cone levels of β-catenin protein. We find that the 3'UTR of β-catenin mRNA is sufficient to direct protein expression to the axon. Taken together these findings point to an important role for β-catenin in post-mitotic neurons during the development of thalamocortical connectivity.

## Methods

### In situ hybridization

Antisense and sense digoxigenin-labelled RNA probes were synthesized using a DIG transcription kit (Roche, UK). IMAGE consortium [74] clone i3156732 was used to generate *β-catenin *probes corresponding to 932 bp of the 3'UTR. In situ hybridation was performed as described previously [75]. Briefly, tissues were fixed in 4% paraformaldehyde, incubated with RNA probes overnight at 70°C in hybdridisation buffer including 50% formamide and 5xSSC, reacted with alkaline phosphatase conjugated anti-digoxigenin antibodies (1:500 at 4°C overnight; Roche, UK) and stained with nitro blue tetrazolium chloride/5-Bromo-4-chloro-3-indolyl phosphate (NBT/BCIP; Roche, UK). Images of stained axons were quantified using ImageJ software to measure the pixel intensity along the length of the axon. To combine data from a sample of cells the position of each pixel was expressed as a percentage of axon length, with 0% at the cell body and 100% at the growth cone, and intensities binned into 5% segments.

### Immunohistochemistry

Tissue was fixed in 4% paraformaldehyde at 4°C, overnight for embryo heads or 1 hr for cultured explants or cells, reacted with rabbit anti-neurofilament (1:500, Affinity, UK) and/or mouse anti-β-catenin (1:1000, 610154 BD Biosciences, UK) antibodies followed by DAB immunohistochemistry using an ENVISION lit (Dako) or fluorescent secondary antibodies Alexa fluor goat anti-mouse 488 and Alexa fluor goat anti-rabbit-568 (1:200; Invitrogen) for immunofluorescence. For GFP detection thalamic cultures were fixed in 2% paraformaldehyde, and processing for GFP immunohistochemistry using rabbit anti-GFP (1:8000; Abcam 290) followed by a goat anti-rabbit-488 nm secondary antibody (1:200; Invitrogen).

### LacZ Histochemistry

Embryonic heads were dissected and fixed overnight at 4°C in LacZ Fix [4% paraformaldehyde, 0.02% NP40, 0.01% sodium deoxycholate, 5 mM EGTA, 2 mM MgCl_2 _in phosphate buffered saline (PBS)]. Heads for thin frozen sections were equilibrated in 30% sucrose/PBS at 4°C, embedded in OCT and sectioned (10 μm) using a cryostat. Sections were collected on poly-L-lysine coated glass slides, rinsed several times in wash buffer (2 mM MgCl_2_, 0.02% NP40, 0.01% sodium deoxycholate in PBS), transferred to LacZ stain (wash buffer supplemented with 5 mM potassium ferricyanide, 5 mM potassium ferrocyanide and 1 mg/ml X-gal), stained for at least 20 hours at 37°C, and counterstained with Nuclear Fast Red.

### Embryonic thalamus culture

(1) Netrin response and in situ hybridisation experiments: E14.5 thalami from CBA embryos were diced to give > 50 small explants/thalamus which were cultured on poly-L-lysine (0.001%, Sigma) and fibronectin (1 mg/ml, Sigma) coated glass coverslips for three days in serum free medium to allow extension of neurites. Cultures were then exposed to Netrin-1 by replacing 40% of the culture medium with fresh culture medium supplemented with Netrin-1 (R&D Systems) to give a final concentration of 400 ng/ml. For Netrin-1 free cultures, 40% of the culture medium was replaced with fresh medium. Cultures were then either processed for β-catenin immunofluorescence or RNA extracted from the whole culture (explant + axons) for qRT-PCR analysis. (2) Thalamic axon plasmid cDNA library: Explant cultures were carried out as described previously [76]. Briefly, 250 μm-thick explants of thalamus were dissected from coronal slices of embryonic day 14.5 (E14.5) TgTP6.3/CBA embryonic mouse brains and arranged on collagen coated inserts (Costar, UK) in serum-free medium and cultured for 2-3 days. (3) Thalamic cell or axon compartment qRT-PCR: Cultures were prepared as in (2) except the CBA embryos were used and serum-free medium was supplemented with 10% fetal calf serum (4) GFP reporter experiment: Dorsal thalamus was dissected from E14.5 CBA mouse embryonic brain and collected in cold oxygenated Earle's Balanced Saline Solution (EBSS). Thalami were mixed with 25 μl of PBS (Phosphate Buffered Saline) containing plasmid DNA (1 mg/ml) and each one was cut into ~25 pieces. Two electrodes were placed in the drop on either side of the pieces and two consecutive square waved pulses applied (70 V for 50 ms 3 times with 900 ms pauses), using a CUY21 EDIT (Sonidel) electroporator. Amperage obtained ranged from 0.02 amps to 0.05 amps. Cold EBSS was immediately added to the dish and the pieces were kept on ice until culturing in a collagen mixture on glass coverslips for 2 days as previously described in [77]. (5) Dissociated thalamic cells were prepared using a Papain Dissociation Kit (Worthington, UK) and cultured on poly-L-lysine (0.001%, Sigma) and fibronectin (1 mg/ml, Sigma) coated glass coverslips for three days in serum free medium.

### Quantifying β-catenin protein in growth cones

Images of growth cones were taken using a Zeiss LSM510 CLSM, Plan-Apochromat ×63/1.4 oil objective zoomed ×3.1. Optical sections of growth cones were taken at a constant stack separation of 0.13 μm and at constant optical gain and laser output. Data were analysed using ImageJ software with each analysis being performed on the entire stack generated from each growth-cone image. Pixel intensities in regions containing no cells or processes were measured to give background values that were used to threshold images of growth cones. For each growth cone, the total number of pixels above background intensity through the stack and the average pixel intensity for all pixels above background intensity were multiplied to give total β-catenin staining intensity. Statistical analysis was done using the Sigmaplot software package.

### Plasmid cDNA library

Samples were cut from thalamic cultures with a sterile scalpel blade using a fluorescence dissecting microscope to visualise GFP-expressing axons, homogenised in RNAzol (Tel-Test, USA) and stored at -20°C before RNA extraction and first strand cDNA synthesis, primed using Not1dT_18 _oligonucleotides, using a reverse transcription kit (Amersham, UK). A differential display polymerase chain reaction (DDPCR) protocol was used to amplify segments of sequence from the cDNA samples. Three 10-mer primers DM1 (ATATCTGGAG), DM2 (CGATCGTGCA), and DM4 (CGGTAACAAG) were used separately, each in combination with an equimolar mixture of clamped oligo-dT primers dT_12 _MM (where M is G, A, or C) in separate DDPCRs to amplify a pool of products from each cDNA sample. ^35^S-dATP was included in the DDPCR reactions to allow visualisation of products on a polyacrylamide gel. DDPCR products produced from each of the cDNA sample types were pooled, cleaned using a GenElute PCR cleanup kit (Sigma, UK), ligated into pGEMTeasy (Promega, UK) and transformed into XL10-Gold Ultracompetent cells (Stratagene, USA); ampicillin resistant colonies were selected. Clones were picked at random and their DNA was sequenced.

### Quantitative PCR

Quantitative RT-PCR experiments were carried out on cDNA samples from three different explant cultures, obtained as described above, using Quantitect Sybr Green PCR kits (Qiagen, UK). Primers were designed using PerlPrimer [78] and all were intron-spanning, except those for 18S, which has only one exon. Standard curves plotting quantities of product generated with each primer pair against numbers of cycles at threshold for a series of dilutions of the starting samples all had *r^2 ^*values of 0.95 or greater [79]. The threshold was chosen as early in the exponential phase as possible to minimize differences due to variation in efficiency between different primer pairs. Analyses of melting curves confirmed that only one product was amplified by each primer pair. The abundance of each cDNA species in each sample was calculated using Opticon software by comparing the PCR reaction kinetics between the sample and a dilution series of a standard cDNA pool produced from E14.5 dorsal thalamic tissue. Oligonucleotide primer sequences were as follows: *Rn18s*, 5'-TCAGTTATGGTTCCTTTGGT-3'/5'-CGAAAGTTGATAGGGCAGAC-3'; *Actb*, 5'-CACCACACCTTCTACAATGAG-3'/5'-GTCTCAAACATGATCTGGGTC-3';

*Ctnnb1*, 5'-CTGCTCATCCCACTAATGTC-3'/5'-CTTTATTAACTACCACCTGGTCCT-3'; *Syt13*, 5'-CAGAAGTCATCAACTACGCA-3'/5'-TCCTCAACTACACCGTTCTG-3'; *Rps3*, 5'-CAAGAAGAGGAAGTTTGTAGCTG-3'/5'-CCCAAGAACATTCTGTGTCC-3'; *Rala*, 5'-TGTACGACGAGTTTGTAGAG-3'/5'-GATCTGACTTGTTACCAACC-3'; *Rtn1*, 5'-GAGCAGATCCAGAAGTACAC-3'/5'-GAAACCACAGCCATAAGCAG-3'; *Tuba1a*, 5'-CAGATGCCAAGTGACAAGAC-3'/5'-GTGCGAACTTCATCGATGAC-3'; *Mtap2*, 5'-CTTCGGCTTATTAACCAACCA-3'/5'-GGCTGTCAATCTTCACATTACC-3'; GAPDH: 5'-GGGTGTGAACCACGAGAAAT-3'/5'-CCTTCCACAATGCCAAAGTT-3'; *LacZ *5'-CGAAATCCCGAATCTCTATCGTGC-3'/5'-GATCATCGGTCAGACGATTCATTG-3'; *EphA4*, 5'-CCATCAAAATGGACCGGTAT-3'/5'-CATCTGCTGCATCTGGGTTC-3'; *L1*, 5'-GTTCATCGCCTTTGTCAGC-3'/5'-CCG AAG GTC TCG TCT TTC AT-3'.

### Plasmid construction

The CAGG promoter element [69] from pTP6 [70] and myrd1EGFP cDNA [53] were inserted between the *SalI/EcoRI *and *EcoRI/PstI *restriction sites of pBluescriptKS (Stratagene) respectively to generate pTP7 in which a *CAGG:myrd1EGFP *cassette lacking mammalian polyadenylation signals is immediately 5' to a unique *Not1 *restriction site. A partial clone of the *β-catenin *gene was recovered from mouse genomic DNA by sequential PCR using primer pairs 5'-CCCAGCTACCGTTCTTTTCA-3' & 5'-GAGCTGAAGGGCTGGTTA CA-3' followed by 5'-ATGGACCCTATGATGGAG CA-3' & 5'-TCAGCCCTTTGGTCAGAAGT-3' to generate a 1396 bp *β-catenin *sequence spanning from 100 bp 5' to the translation stop codon to 200 bp 3' to the polyadenylation signal so including the entire 3'UTR and sequence 3' to the polyadenylation signal. pKR6 was generated by inserting this 1396 bp sequence into the pTP7 *Not1 *site. pKR7 was generated using PCR primers 5'-GCGGCCGCTGCTTCAACAGATGCGGTTA-3' & 5'-GAGCTCGTTTGCCTGGGTTTTGATGT-3' to make a 287 bp truncated fragment of the 1396 bp *β-catenin *sequence spanning from 80 bp 5' to the polyadenylation signal to 200 bp 3'to the polyadenylation signal and inserting this 3' to the pTP7 *Not1 *site. Plasmid construction was performed using standard restriction enzyme digest and T4 DNA ligation and PCR products were subcloned into pGEMTeasy (Promega). All PCR was performed with the high fidelity polymerase *PfuTurbo *(Stratagene) and plasmids verified by sequencing (MWG Biotech).

### GFP reporter analysis

GFP stained material was imaged with a Zeiss Axiovert LSM510 CLSM (Carl Zeiss Ltd, Germany). Myrd1EGFP reporter expression from pKR6 and pKR7 or tauGFP reporter expression from pTP6 [70] in the axon was quantified by measuring the distance which GFP immunofluorescence could be traced from the cell body along the axon. Cell body myrd1EGFP expression was quantified by measuring the intensity in a freehand selection drawn round the cell body and subtracting the background level measured in an area of the explant not expressing GFP. ImageJ was used for measurements and the Sigmaplot software package for statistical analysis.

## Abbreviations

qRT-PCR: quantitative reverse transcription polymerase chain reaction; cDNA: complementary DNA; mRNA: messenger RNA; BLAST: basic local alignment search tool; GFP: green fluorescent protein; UTR: untranslated region; ORF: open reading frame; PBS: phosphate buffered saline; E15.5: embryonic day 15.5; T: thalamus; pT: pre-thalamus; ic: internal capsule; vtel: ventral telencephalon; cc: cerebral cortex; E: explant; cb: cell body; a: axon; gc: growth cone.

## Competing interests

The authors declare that they have no competing interests.

## Authors' contributions

TP prepared the cDNA library, constructed plasmids, performed immunohistochemistry, participated in the design of the study, and drafted the manuscript. JWD performed the qRT-PCR and in situ hybridisation and participated in the design of the study. TJN performed the Netrin-1 experiments. CR performed the GFP reporter assays. KR constructed plasmids and performed sequence analysis. DM helped prepare the cDNA library. MC participated in the design of the study. JOM and DJP participated in the design of the study and helped to draft the manuscript. All authors read and approved the final manuscript.
